# Severe acute respiratory syndrome coronavirus 2 reinfection in a coronavirus disease 2019 recovered young adult: a case report

**DOI:** 10.1186/s13256-021-02965-5

**Published:** 2021-07-16

**Authors:** Hussein Awada, Hasan Nassereldine, Adel Hajj Ali

**Affiliations:** Faculty of Medicine, American University of Beirut, Beirut, Lebanon

**Keywords:** SARS CoV-2, COVID-19, Reinfection, Immune response

## Abstract

**Background:**

Coronavirus disease 2019 has been a public health threat and a worldwide emergency for more than a year. Unfortunately, many questions concerning the pathophysiology, management, and long-term side effects remain unanswered, and novel aspects of the disease keep on emerging. Of concern to healthcare providers are the recent reported cases of reinfection. Serum coronavirus disease 2019 antibodies have been detected within a few days after onset of the disease. However, it remains unclear whether this immune response is universal, or whether it can lead to latent immunity.

**Case presentation:**

A previously healthy 27-year-old white man presented with fever, chills, back pain, and other constitutional symptoms, 2 days after being exposed to coronavirus disease 2019 positive patients. His severe acute respiratory syndrome coronavirus 2 polymerase chain reaction was positive, and his symptoms resolved over the next 2 weeks. One month after a confirmatory negative severe acute respiratory syndrome coronavirus 2 polymerase chain reaction, he was found to be ineligible for plasma donation as his anti-severe acute respiratory syndrome coronavirus 2 serology was negative. The patient redeveloped symptoms similar to his first infection 3 weeks after the negative serology test. He and his wife both tested positive via polymerase chain reaction. Their symptoms resolved over the next few days, and they had a negative polymerase chain reaction test 10 days after the positive polymerase chain reaction.

**Conclusion:**

While studies showed that anti-severe acute respiratory syndrome coronavirus 2 immunoglobulins start to develop early after infection, our healthy young patient’s immune system failed to mount latent immunity against the virus. This left him, especially amid widespread social and medical misconceptions, vulnerable to reinfection by severe acute respiratory syndrome coronavirus 2. Our case disputes the timelines for immune response that were set and supported by research studies. Our case also raises questions regarding prioritizing vaccinating other individuals over those with prior infection.

## Introduction

Coronavirus disease 2019 (COVID-19), caused by the severe acute respiratory syndrome coronavirus 2 (SARS-CoV-2), first appeared in Wuhan, China, in December 2019 [1]. Since then, COVID-19 has rapidly spread across the world, and it has been declared as a global pandemic by the World Health Organization (WHO) [1]. In Lebanon, the first case was documented on 21 February 2020, and to date, more than 280,000 cases and 2300 deaths have been recorded [2, 3]. While the majority of patients recover from COVID-19 worldwide, a growing concern about reinfection has been developing due to the increasing number of recovered patients who have been reported to have tested positive again [4, 5]. Some of these reports have been contested as false positives, while others have been attributed to quick reexposure before a protective immune response has been mounted. Nevertheless, many studies did demonstrate that recovered COVID-19 patients do develop antibodies against SARS-CoV-2 [6–8]. While there is no clear evidence whether all patients do develop protective latent immunity or how long may it last, several studies emphasized the improbability of reinfection due to a postinfection immunity that is at least short and temporary [4, 9, 10]. In this paper, we report a unique case of a patient, from Lebanon, who recovered from COVID-19 before getting reinfected with SARS-CoV-2 within 2 months of initial recovery.

## Case

**Fig. 1:**
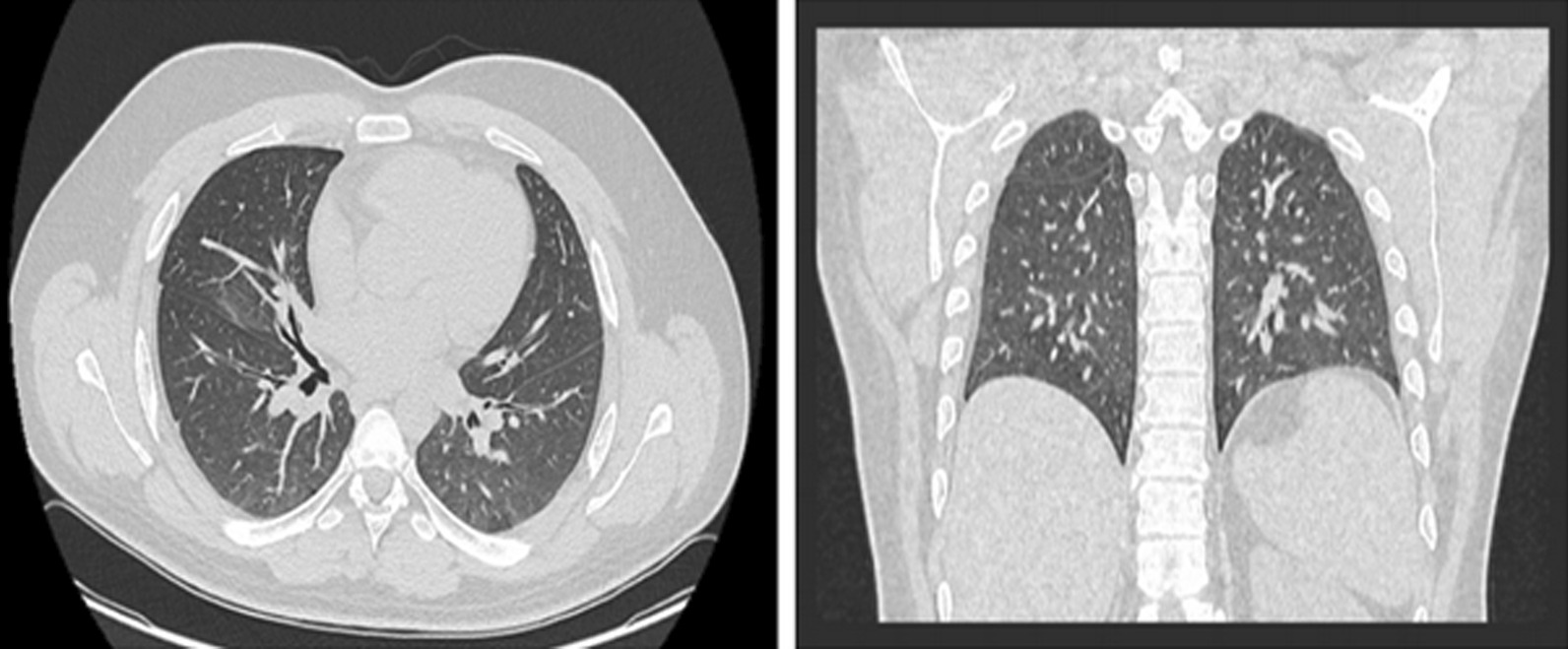
Transverse and coronal sections CT scan of the chest showing emphysematous changes

On 22 November 2020, a 27-year-old white man developed a fever, measured orally as 40 °C (104 F), in addition to chills, diffuse arthralgia, myalgia, headache, and back pain. His other vital signs were all within normal ranges [heart rate 69 beats per minute, blood pressure (BP) 118/76 mmHg, SpO_2_ 97%]. He was previously healthy, with no history of smoking or alcohol intake, not on any medications, and with a family history that was negative for chronic diseases. He worked as a policeman. The patient had contact with two COVID-19-positive patients and thus was instructed by his family physician to undergo a SARS CoV-2 real-time polymerase chain reaction (RT-PCR) after developing these symptoms. The patient was awake, alert, and oriented, and examination of the ears and throat showed no erythema or exudates in the tympanic membranes or tonsils. He had clear breath sounds bilaterally with no crackles, and cardiac auscultation suggested a regular rate and rhythm with no abnormal murmurs. He did not have any enlarged or painful lymph nodes. There were no other findings on physical and neurological examination. A nasopharyngeal swab was taken, and the PCR result came back positive. He underwent routine laboratory testing including complete blood count, fibrinogen, D-dimer, basic metabolic panel, and C-reactive protein, all of which were all within normal levels (red blood cells 4.5 × 10^6^/μL, hemoglobin 13.8 g/dL, white blood cells 6.69 × 10^3^/μL, fibrinogen 3.07 g/L, D-dimer 189 ng/mL, creatinine 0.99 mg/dL, sodium 137 mEq/L, potassium 4.0 mEq/L, C-reactive protein 3.7 mg/L, ferritin 143.5 ng/mL). However, a computed tomography (CT) scan of the chest without contrast revealed the presence of mild emphysematous changes (Fig. 1). Accordingly, the patient was instructed to self-quarantine at his house. During the disease period, the patient developed watery diarrhea on day 4 and anosmia on day 6. The fever subsided 3 days after symptom onset, and the patient was completely asymptomatic 8 days after the positive PCR with the exception of anosmia. The patient required only oral Panadol (paracetamol) 1000 mg every 6 hours for pain and fever for 3 days. The patient never developed any cough or shortness of breath and, hence, is considered to have had a mild infection. On day 13, the patient redid the PCR test, which turned out to be negative.

One month after the infection (26 December 2020), the patient’s COVID serology was tested for the possibility of plasma donation. His anti-SARS-CoV-2 IgG levels were found to be low (4.32 RU/mL) (reference range: negative < 8, borderline 8–11, positive > 11 RU/mL). He was informed that he did not develop sufficient immunity and so was unable to donate plasma.

**Fig. 2:**
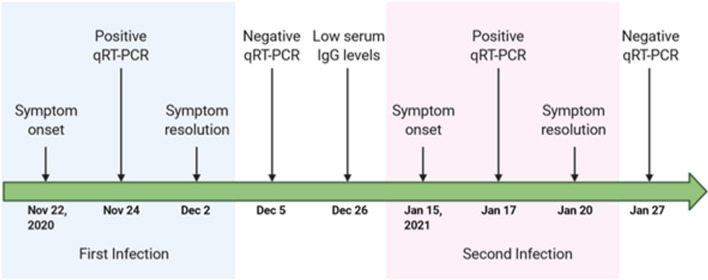
Timeline of symptom onset, patient diagnosis, and recovery

On 17 January 2021, the patient developed fever again, and it was orally measured as 38 °C (100.4 F). He also had a new headache. His heart rate was 68 beats per minute, blood pressure 121/72 mmHg, and SpO_2_ 98%. He was awake, alert, and oriented, and he had clear breath sounds over both of his lungs. The remainder of his physical and neurological examinations was normal. A day prior, the patient’s wife had developed fever, chills, and diffuse myalgias. Both of them had contact with a confirmed COVID-19 patient a few days prior. The two individuals underwent SARS CoV-2 RT-PCR; both of them had positive tests, and they were instructed to self-quarantine at home. It should be noted that SARS-CoV-2 strain-specific PCR kits were not available in Lebanon at the time given the lack of widespread circulation of other strains in the country. The rapid influenza antigen and viral respiratory panel tests were negative in both patients. The patient’s fever subsided 1 day after its onset without any antipyretic use. The patient repeated the PCR test 10 days after the last one, and it was found to be negative. He did not require any medications during the second infection. The timeline of the events up to this point is presented in Fig. 2.

The patient repeated the serum anti-SARS-CoV-2 IgG test on 22 February 2021, and the test came back positive this time (38.4 RU/mL). Hence, he was able to donate plasma this time.

The patient was last seen on 31 May 2021, and he reported being in good health since his last recovery. His physical examination was normal at the time.

## Discussion

In contrast to the literature, our case represents a patient who was confirmed to have recovered from COVID-19 through a negative qRT-PCR test, did not generate sufficient COVID-19 immunoglobulin response by the first month postinfection, and was reinfected by SARS-CoV-2 2 months following his complete recovery. This case represents a challenge to the literature given the fact that our patient’s immunoglobulin levels and the timing of the second positive qRT-PCR test do not match the viral shedding and immunoglobulin durations reported by the studies out there. Hence, it provides evidence that even healthy young adults may fail to mount a protective immune response against SARS-CoV-2 following recovery and thus, may be vulnerable to reinfection by the same virus. The occurrence of mild symptoms at both episodes in our patient is due to the general noninvasive pattern of the disease in the younger population. In such circumstances, the immune response may be faint, and hence, the patient may fail to develop sufficient immunity [8].

More than a year since the outbreak of COVID-19, there is still no consensus in the literature regarding the issue of reinfection with SARS-CoV-2. Yet, reports from around the world do show evidence that reinfection is indeed emerging [11–14]. The gold standard for the diagnosis of COVID-19 is via real-time reverse transcription polymerase chain reaction (RT-PCR) [1]. However, many reports of positive qRT-PCR tests recorded only a few days following confirmed recovery (via negative qRT-PCR tests or an appropriate isolation period) have been dismissed as not reinfection, rather being attributed to other possible explanations [15, 16]. These explanations include (1) possible qRT-PCR false negatives at the time of discharge, (2) persistent viral shedding and increased replication because of early treatment discontinuation following clinical symptoms improvement, and (3) dead viruses and remnant genomic fragments [17–19].

What further increased the incredulity of dubious cases was that they presented quickly—within a few days—following recovery and short of the normal duration needed for the body to develop latent immunity [5]. In a study by Zhao *et al*., it was shown that COVID-19 IgM and IgG levels require a median of 12 and 14 days, respectively, following symptoms onset to become detectable in patients’ blood [8]. Another study indicated that the COVID-19 antibodies and their protective effects could last about 40 days, after which there exists a possibility for reinfection [5]. Furthermore, studies showed that viral shedding lasts 8–37 days in general, with a median of 20 days [20]. While we acknowledge that viral shedding can last up to 3 months, with the longest recorded period in a survivor being 104 days, most studies have shown that persistent viral shedding beyond 1 month after symptom onset is extremely rare [21–25]. Thus, it would be extremely unlikely for our patient to have persistent shedding at the 2-month mark.

Genomic sequencing is the ultimate test that can distinguish between viral shedding and viral reinfection by the same/different strain [26]. Unfortunately, genomic testing for phenotypic characterization was not yet available in Lebanon. Nevertheless, the patient’s wife did not develop symptoms until she was exposed to another COVID-19 patient, suggesting that her husband was also reinfected at that time rather than having viral shedding, which would have infected her much earlier. This narrative of reinfection is further reinforced by the fact that our patient was able to develop immunity only after the second episode. Moreover, at the time of the first infection, there was no evidence of the presence of a SARS-CoV strain other than the original (first identified in Wuhan) circulating in Lebanon, and by the time of the second infection, only the Alpha variant (of Pango lineage B.1.1.7, identified first in the UK) was further recorded but to a very limited extent compared with the spread of the original strain [27, 28].

Further studies are needed to determine the factors that may hinder the immune system from mounting an adequate response to protect from subsequent SARS-CoV-2 reinfections. In addition, routine serologic screening of recovered patients may be of importance in stratifying the risk of reinfection by SARS-CoV-2 in these patients. This is especially important given the false impression held by some patients about the protective personal and public health measures being expendable after recovery, as well as the false belief that all recovered patients would not need to receive a SARS-CoV-2 vaccine afterwards.

## Conclusion

SARS-CoV-2 reinfections remain to be fully clarified in the literature. This case shows that healthy young adults may fail to develop latent immunity and, as a result, could be prone to reinfection. Therefore, personal and public health protective measures remain of utmost importance. Additional research is also required to understand the immune response to this virus.

## Data Availability

Copies of SARS-CoV-2 PCR test results, laboratory tests, and the CT scan are available upon request by the Editor-in-Chief of this journal (not uploaded upon submission for confidentiality purposes).

## Abbreviations

COVID-19: Coronavirus disease 2019
SARS-CoV-2: Severe acute respiratory syndrome coronavirus 2
WHO: World Health Organization
qRT-PCR: Real-time quantitative reverse transcription polymerase chain reaction
IgG: Immunoglobulin G
IgM: Immunoglobulin M
CT: Computed tomography
